# Hypertension and Life-Threatening Bleeding in Children with Relapsed Acute Myeloblastic Leukemia Treated with FLT3 Inhibitors

**DOI:** 10.4274/tjh.2014.0250

**Published:** 2015-08-01

**Authors:** Deniz Yılmaz Karapınar, Nihal Karadaş, Zühal Önder Siviş, Can Balkan, Kaan Kavaklı, Yeşim Aydınok

**Affiliations:** 1 Ege University Faculty of Medicine, Department of Pediatric Hematology, İzmir, Turkey

**Keywords:** Acute myeloblastic leukemia, FLT3, Sorafenib, Sunitinib, children, D835Y mutation

## Abstract

Multikinaz inhibitörleri ile deneyim, özellikle çocuklarda çok sınırlıdır. Burada, biri FMS-like tirozin kinaz 3-ITD ve diğeri tek baz mutasyonu (D835Y) taşıyan nüks akut miyeloblastik lösemi (AML) tanılı iki hastadan edindiğimiz deneyimi sunmak istedik. Her iki hasta sorafenib (biri 19, diğeri 42 gün olmak üzere) tedavisini klofarabin içeren yoğun kemoterapi protokolü ile birlikte aldı. D835Y mutasyonu bulunan hasta daha sonra 20 gün sunitinib ile tedaviye devam etti. Her iki hastada da ağır pansitopeni, hipertansiyon, yaşamı tehdit eden gastrointestinal sistem kanaması ve pulmoner kanama gelişti. Her iki hastanın kemik iliğinde ağır bir aplazi sağlanmış olsa da blastik infiltrasyon görülmeden nötropenik sepsisle hastalar kaybedildi.

## ABSTRACT

Experiences with new multikinase inhibitors are limited, especially in children. In this report we summarize our experience with 2 patients with relapsed acute myeloblastic leukemia (AML), one with FMS-like tyrosine kinase-3-internal tandem duplication mutation and the other with a single base mutation (D835Y). Both patients received sorafenib, one for 19 days and the other for 42 days, with clofarabine-including chemotherapy. One additionally received sunitinib for a total of 20 days. Both patients developed severe pancytopenia, hypertension, life-threatening bleedings from the gastrointestinal system, and, finally, intrapulmonary hemorrhage. Although both reached severe aplasia of the bone marrow without blastic infiltration, death occurred with neutropenic sepsis.

## INTRODUCTION

The FMS-like tyrosine kinase-3 (FLT3) gene is mutated in approximately 30% of acute myeloblastic leukemia (AML) cases in adults and 10%-15% in children, especially those with normal karyotypes [1,2]. The most common mutation is internal tandem duplication (ITD). Single base mutations may also be seen, most commonly resulting in a substitution of aspartic acid with tyrosine or less commonly a histidine at residue 835 in the tyrosine kinase domain (D835). These mutations result in constitutive activation of the FLT3 receptor, hence downstream of some pathways [1,3,4]. They cause enhanced proliferation and reduced apoptosis of the myeloblasts, which contributes to leukemogenesis. Generally they are associated with leukocytosis, normal cytogenetics, lower remission and higher relapse rate, and worse survival [1,2,3,5,6]. Tyrosine kinase inhibitors (TKIs) work as competitive inhibitors of ATP, binding to its pocket in the kinase domain, and are used for targeted therapy to control tumor growth and angiogenesis [3,5,6]. Experiences with TKIs in children are restricted, showing some benefits with acceptable toxicities [7,8,9,10]. Here we report 2 pediatric relapsed AML patients treated with TKIs.

## CASE PRESENTATION

Patient 1 was a 2-year-old girl diagnosed with AML after presenting with hyperleukocytosis and normal cytogenetics. She received chemotherapy according to the AML-BFM 2004 protocol and reached remission after the first course of induction therapy. No donor was available for stem cell transplantation and she developed relapse 5 months after remission was achieved. Combined chemotherapy including idarubicin (12 mg/m2/day for 3 days), fludarabine (30 mg/m2/day for 4 days), and cytarabine (2 g/m2/day for 4 days) was administered. Bone marrow aspiration (BMA) on day 28 showed 90% blastic cell infiltration. In retrospective analyses of bone marrow samples, FLT3-D835Y was found to be positive at initial diagnosis. Although it disappeared after the first course of induction chemotherapy, it was found to be positive again at relapse and 28 days after IDA-FLA treatment. After getting permission from the Turkish Republic Ministry of Health and from the patient’s family, sorafenib (200 mg/m2/day) was started as a salvage therapy. To prevent hand-foot-skin toxicities, protective measures were taken, such as prophylactic extensive moisturizing by local emollients to the whole body, and the patient was advised to wear soft, thin, cotton gloves and socks on her hands and feet. Five days later combined chemotherapy including clofarabine (40 mg/m2/day for 5 days) and cytarabine (1 g/m2/day for 5 days) was added to the sorafenib. One week later she had complaints of stomachache and developed hypertension. Although she had very severe pancytopenia, BMA at 28 days of chemotherapy did not show any decrease in the percentage of blastic cells. Therefore, at 32 days, sorafenib was switched to sunitinib (15 mg/m2/day). Three days after sunitinib treatment was started the hypertension increased and abdominal discomfort became prominent; several X-ray and ultrasonographic evaluations were performed and no obvious cause was identified. Electrolyte imbalance was seen (hypocalcaemia and hypokalemia; both required high amounts of intravenous replacement). She had gastrointestinal system (GIS) hemorrhage with normal prothrombin time (PT), activated partial thromboplastin time (APTT), and fibrinogen levels at day 7 of sunitinib treatment. It was managed with intensive support and the dose of sunitinib was lowered to 7.5 mg/m2/day. However, symptoms did not resolve and severe pancytopenia persisted with a white blood cell count below 100/mm3 and transfusion dependency. Severe hypertension was under control only after triple antihypertensive agents were added to the treatment. Very severe aplasia without blastic infiltration was seen in BMA at day 18 of sunitinib treatment. She again developed a severe upper GIS hemorrhage on day 19. Therefore, sunitinib was stopped at the 20th day. In spite of aggressive and prompt support with fresh frozen plasma and platelet and erythrocyte transfusions, she had a massive pulmonary hemorrhage, requiring intubation and mechanical ventilation. She expired with neutropenic sepsis 2 days after sunitinib was stopped. Informed consent was obtained.

Patient 2 was a 12-year-old boy diagnosed with AML after presenting with hyperleukocytosis and normal cytogenetics. He received chemotherapy according to the AML-BFM 2004 protocol and achieved remission after the second course of induction therapy. He developed relapsed AML 4 months after first remission was achieved. IDA-FLA was administered. His BMA at day 28 showed 75% blastic cell infiltration. In retrospective analyses of bone marrow samples, FLT3-ITD was found to be positive at initial diagnosis. Although it disappeared after the second course of induction chemotherapy, it was found to be positive again at relapse. No hematological or molecular remission was seen after IDA-FLA treatment. On day 15, BMA showed severe aplasia. After the same formal permission procedure was followed and prophylactic measures were taken to prevent severe skin toxicities, sorafenib was started at a dose of 2x200 mg/m2/day on day 21 as salvage treatment. However, on day 28, repeated BMA showed 40% blastic cells. On day 29, 8 days after sorafenib was started, he developed hypertension and required antihypertensive therapy. Second-line relapse treatment with clofarabine (40 mg/m2/day for 5 days) and cytarabine (1 g/m2/day for 5 days) was started. He complained about severe muscle and bone pain and stomachache. Very severe pancytopenia occurred. BMA 15 days after second-line treatment was negative for FLT3-ITD mutation; he had then been receiving sorafenib for 42 days. After a total of 45 days of treatment with sorafenib he developed severe hypertension, abnormal renal function (urea: 176 mg/dL, creatinine: 5.9 mg/dL), severe metabolic acidosis, and intraocular and severe mouth bleeding. He was still in deep pancytopenia (WBC: 0.016x109/L, Hb: 49 g/L, Hct: 13% PLT: 17.7x109/L). Fibrinogen levels and PT and APTT were in the normal ranges. Sorafenib was ceased and intensive support with aggressive blood product supplementation was given. Because renal functions progressively worsened, renal replacement therapy with hemodialysis was started. He developed pulmonary hemorrhage; intensive support including platelet suspension, fresh frozen plasma, and recombinant factor VIIa was given. He developed neutropenic sepsis and died 12 days after cessation of sorafenib.

## DISCUSSION AND REVIEW OF THE LITERATURE

FLT3-mutated AML patients respond poorly to conventional chemotherapy and have worse prognosis. Current studies put them into high-risk groups and they are candidates for hematopoietic stem cell transplantation. Sunitinib and sorafenib are multikinase inhibitors and were approved for treatment of renal cell and hepatocellular carcinoma. Some small case series and trials with sunitinib and especially with sorafenib alone or in combination with chemotherapy for treatment of refractory or relapsed AML for salvage therapy have been published [4,7,8,9,10,11,12,13,14]. Some of them showed that hematological remission may be achieved with acceptable toxicity profiles [4,9,10].

In this report we summarize our experience in 2 patients with relapsed AML, one with FLT3-ITD mutation and the other with D835Y. Both patients received idarubicin, fludarabine, and cytarabine combination therapy after hematological relapse occurred. Hematological remission could not be achieved. They were given TKIs with combined chemotherapy and reached severe aplasia with clearance of blastic cells. It was shown that sorafenib inhibits FLT3-ITD more potently than FLT3-D835Y, while sunitinib is equally effective against both mutant forms of FLT3 [13]. The patient with D835Y mutation thus received sunitinib since no response was seen after sorafenib.

The most common toxicities of TKIs are reported as reversible skin rash, hand-foot-skin reaction, diarrhea, abdominal pain, mild myelosuppression, and electrolyte abnormalities [5,7,9,10]. Children with leukemia who received sorafenib in combination with other agents experienced grade 2 or 3 hand-foot-skin reactions very frequently, at rates of up to 100% [5,7,9,10]. In adults, one-third of patients required dose reductions due to skin toxicity or diarrhea. Hypertension was infrequently reported in children, despite being very common in adults [7,9,10,11,12]. In these presented cases, most probably the prophylactic measures prevented skin toxicities, but the patients had very severe hypertension, which possibly contributed to the life-threatening bleedings.

The effects of TKIs are related to active N-oxide metabolites [9]. These metabolites might also be responsible for the toxic effects. A much higher rate of conversion of TKIs to active metabolites was shown in children than in adults by pharmacokinetic studies [9,10]. Thus, higher exposure to the metabolites may contribute to increased toxicity among children.

In our patients, persistent very severe pancytopenia did not allow us to assess remission status. Both of the patients were transfusion-dependent and intensively supported. Both patients died with neutropenic sepsis after major pulmonary hemorrhage.

The complications observed in our patients suggest that efficacy of TKIs may be limited by their toxicities. In particular, myelosuppression, severe hypertension, and bleeding problems had very significant effects on the continuation of therapy and resulted in death.

Experiences with new multikinase inhibitors such as sorafenib or sunitinib are limited, especially in childhood AML. The requirement for prospective randomized studies to determine the safety and efficacy of these drugs is clear.
